# REMIL-IBD: Region-filtered multiple instance learning for interpretable slide-level grading of inflammatory bowel disease

**DOI:** 10.1016/j.jpi.2026.100674

**Published:** 2026-05-14

**Authors:** Ankana Banerjee, Ole Gunnar Aasprong, Emiel A.M. Janssen, Mark van der Giezen, Tore Grimstad, Kjersti Engan

**Affiliations:** a Department of Chemistry, Bioscience and Environmental Engineering, University of Stavanger, Stavanger, Norway; b Department of Pathology, Stavanger University Hospital, Stavanger, Norway; c Unit of Gastroenterology, Department of Internal Medicine, Stavanger University Hospital, Stavanger, Norway; d Department of Electrical Engineering and Computer Science, University of Stavanger, Stavanger, Norway; e Research Department, Stavanger University Hospital, Stavanger, Norway; f Natural Resources Institute, University of Greenwich, Chatham, UK; g Institute for Biomedicine and Glycomics, Griflth University, Queensland, Australia; h Department of Clinical Science, University of Bergen, Bergen, Norway

**Keywords:** Computational pathology, Inflammatory bowel disease, Whole-slide classification, Multiple instance learning, Nancy index, Histology foundation model

## Abstract

Histopathological assessment of inflammatory bowel disease (IBD), including ulcerative colitis and Crohn's disease, is essential for diagnosis, disease monitoring, and treatment planning. Standardized scoring systems such as the Nancy Histological Index (NHI) provide structured measures of disease activity; however, reproducibility can vary across observers, particularly for intermediate grades of inflammation. As digital pathology continues to expand in clinical practice, it opens new possibilities for automated, consistent, and interpretable assessment of histological disease activity using routine clinical data.

In this study, we propose REMIL-IBD (Region-Filtered Embedding-based Multiple Instance Learning), a weakly supervised framework for automated grading of inflammation directly from whole-slide images of hematoxylin and eosin-stained biopsies. The proposed approach leverages pretrained histology foundation models within an attention-based learning framework to identify diagnostically relevant tissue regions and generate interpretable slide-level predictions from routine clinical data. By relying only on slide-level labels, the method reduces the need for detailed region-level annotations and supports practical deployment in real-world clinical settings.

We evaluated three REMIL-IBD variants using publicly available foundation models (UNI, Virchow2, and Cerberus) across conventional five-class NHI grading and clinically motivated three-class disease activity groupings. The proposed framework achieved accuracies of approximately 82–84% in three-class classification tasks, with particularly strong discrimination between histological remission and severe active disease. Region filtering enables the extraction of quantitative tissue features and gives improved classification performance for higher-severity inflammation grades, while introducing additional preprocessing time per slide.

The results demonstrate that pretrained histology foundation models can enable reliable, interpretable grading of IBD inflammation using lightweight downstream models and limited annotation. The REMIL-IBD framework provides a scalable and clinically relevant approach for automated histopathological assessment of IBD in digital pathology workflows.

## Introduction

Inflammatory bowel disease (IBD), encompassing Crohn's disease (CD) and ulcerative colitis (UC), is a chronic gastrointestinal condition affecting an estimated 7 million people worldwide, with Scandinavia among the highest-incidence regions.^2^, ^3^, ^4^, ^5^ Although no curative treatment exists, current therapies can induce and maintain remission. As a result, IBD is increasingly managed as a chronic, lifelong condition, placing a substantial burden on healthcare systems, and increasing the demand for timely and reproducible clinical assessment.

Histopathological evaluation is central to IBD diagnosis, subclassification, and treatment monitoring, as clinical symptoms and endoscopic appearance alone overlap with other gastrointestinal disorders and are not sufficiently specific on their own.^6^ Despite the diagnostic complexity of IBD subtypes, as the disease progresses, they exhibit shared recognizable histological hallmarks of IBD like neutrophil and eosinophil infiltration, crypt distortion, erosion of the gut epithelial barrier, alongside other chronic inflammatory features and changes. These are systematically graded using validated scoring systems such as the Geboes Score (GS), Robarts histopathological index (RHI), and Nancy histological index (NHI).^7^, ^8^ The NHI is recommended by ECCO for its clarity and ease of use in UC.^9^, ^10^

A key limitation of these scoring systems is subjectivity. Whereas extreme grades show reasonable interobserver agreement, intermediate scores (e.g., NHI 1–2) remain prone to disagreement due to ambiguous thresholds between chronic and acute inflammatory features.^11^ A recent global survey revealed that the descriptive use of non-standardized terms like “more,” “mild,” and “moderate” in clinical practice makes it subject to personal interpretation.^12^

With the increasing adoption of digital pathology in labs, combined with advances in artificial intelligence (AI) and machine learning (ML), the field of computational pathology (Cpath) has emerged as a promising approach to address subjectivity in histopathological assessment and the growing demand for efficient diagnostic workflows.^13^, ^14^, ^15^

Although most Cpath research has traditionally centered on oncological histopathology,^15^, ^16^, ^17^, ^18^, ^19^ growing efforts of Cpath have also been applied to IBD. Evolving from early morphometric analyses and supervised classifiers^20^, ^21^ to sophisticated models that focus on specific features, such as mucin depletion or neutrophil presence, to predict relapse and severity.^9^, ^22^ Recent studies using attention-based multiple instance learning (ABMIL) architectures, and commercial systems like PathAI's IBDExplore (94% accuracy), have achieved pathologist-level accuracy in grading histological activity.^23^, ^24^ As potential preprocessing steps, Cpath literature includes artifact detection,^25^ stain normalization,^26^ and tissue segmentation.^27^ Using tissue segmentation as region-filtering before prognostic predictions has been explored in Fuster et al.^28^

Existing models for IBD often rely on large, private training datasets with heavy region-level annotation, task-specific CNN architectures, or focus on coarse “active vs. inactive disease” binary classifications.^29^, ^30^ Available open-source resources such as CoNIC, IBDome, and IBDColEpi provide hematoxylin and eosin (H&E) images but lack IBD-specific annotations or report only binarized disease activity labels rather than per-slide NHI scores.^31^, ^32^ To the best of our knowledge, slide-level NHI grades are not currently available in the public domain. This scarcity of expert-annotated data forces MIL models to learn from weak slide-level labels across large numbers of patches, many of which may be irrelevant or artifactual, limiting robustness, and generalizability.

To address these limitations, we propose REMIL-IBD (Region-filtered Embedding-based Multiple Instance Learning for IBD grading), a weakly supervised framework for automated NHI grading from H&E-stained biopsy images using only slide-level labels. The framework leverages pretrained histopathology foundation models (HFMs) to generate robust tissue representations without requiring labor-intensive region-level annotations, which is particularly advantageous in settings with limited training data. Region filtering serves a dual purpose: it enables the extraction of quantitative tissue features, such as the extent and distribution of specific tissue types and cell populations, and it identifies diagnostically relevant regions from which embeddings are computed using pretrained foundation models. These embeddings are then integrated into an attention-based MIL approach to produce interpretable slide-level predictions.

The key contributions of this work are: (1) A region-filtering strategy that extracts quantitative tissue features and identifies diagnostically relevant regions. (2) A weakly supervised framework for slide-level NHI grading using frozen pretrained HFMs, with trainable attention-based aggregation that supports interpretable predictions. (3) Evaluation of quantitative tissue features and an alternative biologically motivated NHI regrouping strategy for clinically meaningful assessment.

## Data material

We used samples from the Stavanger University Hospital IBD (SUSI) (NCT01551563) project for this study. SUSI is a single-center observational cohort study initiated in early 2012 to assess clinical course, treatment response, QoL, and fatigue in IBD patients. Newly diagnosed patients aged 16–80 years were eligible for inclusion, provided they had no prior history of IBD, were not pregnant, and were able to provide informed consent. Histological grading using the NHI was performed by experienced gastrointestinal pathologists according to established criteria. The NHI was used as the scoring system for both UC and CD biopsy samples due to the absence of a validated CD-specific histological activity index. The study has ethical approval, and written informed consent was obtained from all participants.

The main study included data from four visits: a baseline at diagnosis (V0), followed by three follow-up visits at 3, 11, and 60 months (V3, V11, and V60). For the experiments in this article, we only included biopsies from the first visit (V0), and patients undergoing colonoscopy and clinical examination, with confirmed diagnosis of IBD collected between 2012 and 2024, resulting in 442 patients.

**Fig. 1:**
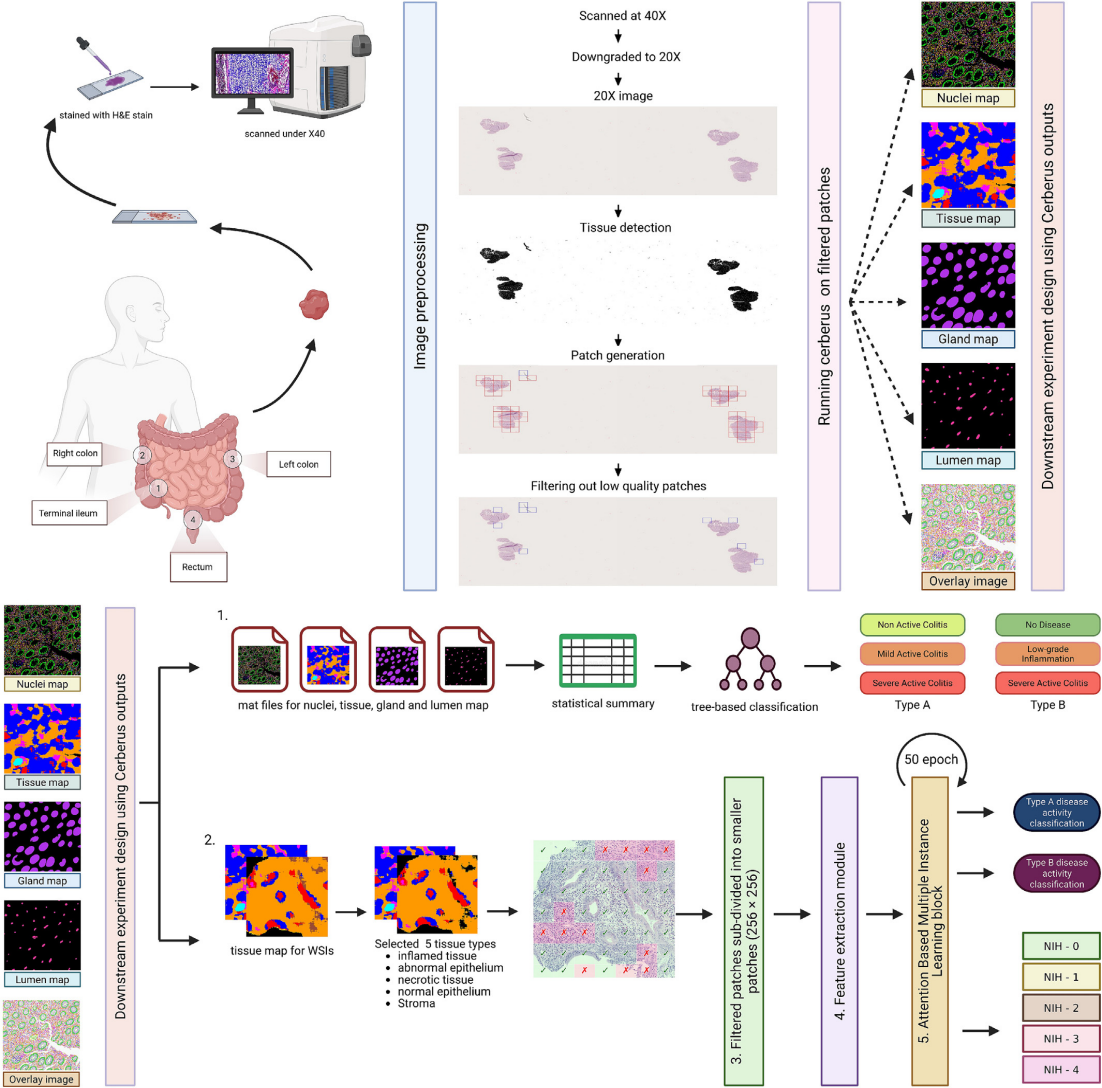
Overview of the study workflow. The top panel shows biopsies were collected from patients enrolled in the study and annotated with Nancy index (NHI) by an expert pathologist. Raw WSI images were processed, patched, filtered for quality, and then run through Cerberus to predict segmentation maps of various histological structures. The bottom panel shows an overview of the classification workflow. 1. From each map, statistical summaries were computed; these aggregated features were used as input for a tree-based classification model, which predicted disease activity level from non-spatial features. 2. Using tissue maps from Cerberus output filtered out irrelevant tissue types, and then patches were extracted using the filtered map from the WSI only where the patches overlapped with regions from the Cerberus maps. 3. Filtered tiles were subdivided into smaller patches. 4. These patches passed through the feature extraction block and patch level features are aggregated to slide level embedding. 5. The embedding is then passed to a classifier layer.

For each patient, biopsies were taken from four distinct anatomical locations according to the standard protocol, as illustrated in Fig. 1 (top left): terminal ileum, right colon, left colon, and rectum from the most representative areas of inflammation or healing. Each of these tissue samples was formalin-fixed in paraffin-embedded (FFPE) blocks, 4 μm H&E-stained sections were digitally scanned at 40X (0.25 μm/pixel) using Hamamatsu NanoZoomer® S60v2.

Technical limitations, patchy inflammation patterns, and the need to prioritize safer or diseased areas resulted in not taking biopsy samples from certain regions for some patients. Similarly, if some areas have multiple lesions, mixed healing patterns, and variable levels of inflammation, clinicians might take more than one biopsy to capture this. A grid layout of the presence–absence schematic is shown in Supplementary Table 1. For patient-level diagnosis and treatment decisions, the slide with the highest inflammation across the four biopsy locations is considered. Severe cases are rare because, in most cases, diagnosis and treatment start before it reaches that severity. Distribution of patient-level maximum NHI scores from our dataset is summarized in Table 1a. As our study focuses on reproducible prediction of NHI irrespective of location using ML, we treated each slide independently, and the raw distribution of NHI scores (0–4) across biopsy locations is summarized in Table 1b.

**Table 1:** (a) Patient-level distribution of maximum NHI scores, where each patient contributes the highest NHI score observed among their four biopsy locations. (b) Slide-level distribution of NHI scores across all individual biopsy locations (terminal ileum, right colon, left colon, rectum; 1743 WSIs), (c) Frequency of different classes across training, validation, and test datasets.

*(a) Patient-level distribution of maximum NHI scores (n = 442 patients)*					

Patient group (max NHI per patient)	NHI-0	NHI-1	NHI-2	NHI-3	NHI-4

No. of patients	6	35	204	145	52

*(b) Slide-level distribution of NHI scores by biopsy location (n = 1743 whole-slide images)*					

Biopsy location (No. of WSIs)	NHI-0	NHI-1	NHI-2	NHI-3	NHI-4

Terminal ileum	270	27	84	6	15
Right colon	223	44	124	45	17
Left colon	197	35	106	70	18
Rectum	108	45	183	113	13

*(c) Frequency of different classes across training, validation, and test datasets*					

Set	NHI-0	NHI-1	NHI-2	NHI-3	NHI-4

Training	46.2%	8.3%	28.6%	13.6%	3.3%
Validation	43.2%	8.9%	28.0%	14.4%	5.4%
Test	46.2%	9.8%	28.9%	11.7%	3.4%

### Data split for training and testing

The dataset was split into training, validation, and test sets at the patient level to prevent patient-level information leakage. The distribution of inflammation grades across the three subsets is summarized in Table 1c.

## Methods

Fig. 1 gives an overview of the method workflow, thoroughly explained in the rest of the section. In the following, we start by proposing an alternative NHI grouping for inflammation grade assessment. Thereafter, the steps of the REMIL-IBD pipeline and its variants are described. Detailed technical background of the HFMs we used and the technical concepts we used in our study has been added in the Appendix A.

### NHI grouping for inflammation grade assessment

Previous work has typically grouped NHI as either 0–2 versus 3–4 or as histological response (0–1) versus active disease (NHI > 1).^29^, ^33^, ^34^, ^35^ Guided by expert pathologist input, we considered that NHI does not always behave as a simple linear severity scale. In particular, although NHI 0 and 1 both lack neutrophilic activity, NHI 1 often reflects healing tissue with residual chronic inflammation rather than truly normal mucosa.

The decision of NHI 1 is also often influenced by the patient's recent clinical history—rather than relying exclusively on unbiased observation of histopathological changes.

We therefore evaluated two three-class grouping strategies in addition to a five-class strategy. In the conventional grouping (Type A), NHI 0–1 was defined as “No Active Colitis,” NHI 2 as “Mild Active Colitis,” and NHI 3–4 as “Severe Active Colitis,” consistent with published definitions and clinical practice.^8^, ^36^, ^37^ In the alternative grouping (Type B), NHI 0 was treated as “No Disease,” NHI 1–2 as “Low-grade Inflammation,” and NHI 3–4 as “Severe Active Colitis.” This alternative grouping was designed to separate true histological remission from persistent inflammatory change. The distribution of each class across location is given in Supplementary Table 2.

### Image preprocessing

An illustration of the preprocessing pipeline is shown in Fig. 1 top panel (center). All raw ndpi whole-slide images (WSIs) were downsampled to X20 objective resolution, because the three FMs used in this study were trained on the same magnification data. The Python library *histoprep* was used for tissue detection. This downscales the WSI to X8 to generate the foreground map, and then the detected tissue areas at the original X20 resolution are divided into 1520 × 1520 × 3-sized tiles. The tile size was kept relatively large to provide context for the tissue segmentation. Tiles with low quality or with less than 5% tissue were removed. The quality cut-off was based on brightness, saturation, hue, and Laplacian variance, and thresholds were determined as described in Ariotta et al.,^38^ with adjusted cut-off values for our dataset.

### Nuclei instance classification and tissue segmentation

Quality-filtered tiles were used as input for the pretrained Cerberus nucleus instance segmentation model to obtain instance segmentation of multiple histological structures, including nuclei, glands, tissue types, and lumen. The Cerberus FM was chosen for tissue segmentation for its domain-specific multitask pretraining on colorectal histological annotations.^39^ The output segmentation maps were checked by an expert pathologist, and based on which type of map gives more accurate segmentation, we selected either the cell or tissue level. Patches that overlapped selected specific IBD-relevant tissue types were then filtered for downstream analysis (described in a later section “Region filtering for ABMIL model input”). No additional pixel normalization was performed for input tile images, as Cerberus automatically normalizes pixel values to 0–255 using min-max scaling during preprocessing. For this study, Cerberus was run with an input patch size of 144 pixels and an output patch size of 36 pixels, providing a good balance between computational speed and accuracy.

The final output for each 1520 × 1520 pixel of RGB tile consisted of separate MATLAB files with matching pixel dimensions as the input, and for each annotation type (nucleus, tissue, gland, and lumen) containing instance, type, and boundary information for the corresponding annotation level. Illustration of the different types of annotation output generated by Cerberus is shown in Fig. 1 top panel (top right). Each pixel in the original image received a distinct annotation for nuclei, tissue, glands, and lumens, along with a unique ID assigned to each instance at each level. For lumen, only an instance map was generated; for tissue, only a type map of tissue classes was generated. Quantitative histological feature tables were prepared for WSIs by collating tile-level Cerberus output.

### FM-region statistics baseline

As illustrated in Fig. 1, the outputs generated by the Cerberus segmentation model serve two complementary purposes in our pipeline. First, they enable the extraction of quantitative statistical features that characterize intestinal tissue architecture across different inflammation severity levels. Second, the same segmentation outputs are used to identify histologically relevant tissue regions that form the basis for downstream embedding extraction in the MIL classification framework.

From the nuclear maps, we quantified the number of each nuclear type, whereas gland maps measured the number of unique glands, average gland circularity, and surface epithelium area. Tissue maps quantified the pixel area corresponding to each tissue type. Combining these maps can provide the distribution of nuclear types across tissues, the epithelial-to-stromal nuclear ratio, and nuclear counts within glands and surface epithelium.

All the quantities were normalized by the corresponding tissue pixel areas for each WSI, defining the statistical features. Although this statistical summary does not retain spatial context, it captures histological hallmarks of IBD progression such as epithelial layer disruption, increased neutrophil infiltration, and glandular depletion. These statistical features were subsequently evaluated using a tree-based classifier to determine whether aggregated features of tissue composition alone are sufficient to differentiate among inflammation severity classes. In addition, the quantitative output provides a human-interpretable slide-level summary that complements and helps explain the performance of the embedding-based ABMIL classification setup.

#### Gradient boost classification

To provide a non-MIL baseline for comparison, we trained gradient boost (GB) classifiers on the FM-Region statistical features. We evaluated the classifiers using both the conventional binary grouping of inactive IBD (NHI < 2) versus active IBD (NHI ≥ 2) and the two multiclass NHI regrouping strategies defined in this study (Type A and Type B). We trained the Classifier using a 5-fold cross-validation and grid search. The hyperparameters for both models were optimized using *keras_tuner*, and the best configurations for each training setting are reported in Supplementary Table 3. Classification performance was assessed using standard evaluation metrics across the tasks.

### REMIL-IBD: Region-filtered embedding-based multiple instance learning for IBD assessment

The proposed REMIL-IBD method is illustrated in Fig. 1, bottom panel line 2. We propose a gated attention-based MIL model as it collates patch-level embeddings into slide-level predictions, and the attention scores offer interpretability. Each slide is represented here as a “bag” of non-overlapping patches.

#### Region filtering for ABMIL model input

Cerberus generated multilevel annotations for each tile, including nuclei, glands, lumen, and tissue compartments. To prepare inputs for the MIL model, we retained tiles containing IBD-relevant tissue classes: abnormal epithelium, inflammatory regions, and debris, which reflect key histopathological changes in IBD, together with stroma and normal epithelium as baseline reference tissue classes. Other categories, such as muscle, mucous, and adipose, were excluded to reduce the data input burden for the ABMIL model and to focus on the most important tissue types for IBD histology. A patch was included if it contained at least 10% of stroma and normal epithelium, and any presence of abnormal epithelium, inflammatory regions, or debris. The selection of these tissue classes was informed by expected histopathological relevance and preliminary exploratory experiments.

Because the foundation models expect smaller RGB inputs, each filtered 1250 × 1250 tile was subdivided into 25 non-overlapping 250 × 250 patches, which were then resized to the input resolution required by each frozen HFM for feature extraction. This approach was used rather than directly resizing the full tile to preserve local morphology while avoiding padding-related edge artifacts and special-case handling during training. Edge and background patches were excluded if they contained ≥60% background to reduce non-informative content and avoid artifacts from tissue borders or empty regions. The resulting filtered patches were used as input to the ABMIL pipeline.

#### Feature extraction

We used three publicly available HFMs for feature extraction: Cerberus, Virchow2, and UNI, resulting in three REMIL-IBD variants (REMIL-IBD-Cerberus, REMIL-IBD-Virchow2, and REMIL-IBD-UNI). These models were selected for their compatibility with weakly supervised learning frameworks and their demonstrated performance in Cpath tasks. Virchow2 was included due to its strong performance in benchmark studies on colorectal cancer,^40^ UNI as a widely adopted general-purpose baseline model in the Cpath literature,^41^, ^42^ and Cerberus for its domain-specific multitask pretraining on colorectal histological annotations.^39^ Although a more recent version (UNI-2h) exists with improvements in target tumor detection and immunohistochemical staining, it had marginal gains on colorectal H&E benchmarks and offered limited benefit for our H&E-only, non-cancerous IBD grading task at a higher computational cost.

**Fig. 2:**
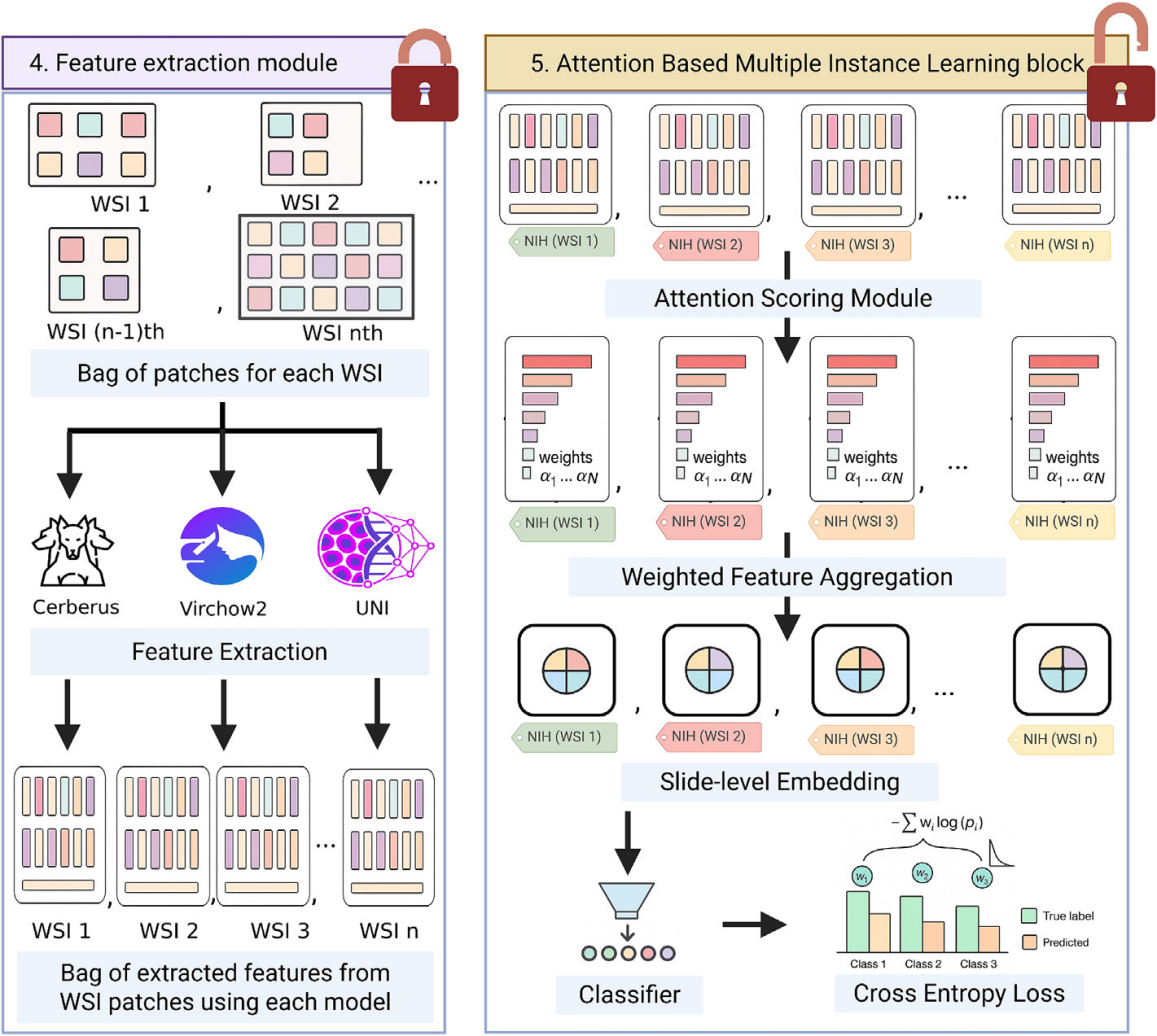
Detailed overview of the REMIL-IBD pipeline. The feature extraction module (left) illustrates patch-wise feature extraction using a frozen histology foundation model (HFM) encoder. The aggregation module (right) shows how patch-level embeddings are combined via an attention mechanism into a slide-level representation, which is passed to a classifier to predict the NHI grade or disease-state label.

Each resized input patch produced 1024-D embeddings for UNI/Cerberus and 2560-D embeddings for Virchow2. These were stored locally, indexed by patient ID and patch base name to preserve patch–patient correspondence. All HFMs were used as frozen feature extractors to reduce the risk of overfitting on our relatively limited dataset size and for computational reasons. An illustration of the feature extraction is seen in Fig. 2 panel 4. The extracted embeddings serve as the input to the attention-based CNN within the ABMIL framework, and ABMIL classification model setup hereafter referred to as REMIL-IBD (details in section “REMIL-IBD classifier”).

#### REMIL-IBD classifier

The ABMIL block of the pipeline is illustrated in Fig. 2 panel 5. This part includes both the learnable attention module and the classifier, and it is trained using the training set. The REMIL-IBD architecture comprises three components:

• Encoder MLP—two fully connected layers with ReLU, dropout (0.3), and layer normalization project patch embeddings to 256 dimensions.
• Gated attention pooling—computes a weight for each patch and forms a weighted sum; then summed into a single-bag representation; padded patches are masked out.
• Classifier—a linear layer mapping the bag representation to three or five inflammation classes.

We compared classification performance for both types of grouping (A and B) using the same REMIL-IBD pipeline, with identical feature extraction, model training, and evaluation metrics. We also trained with the five original NHI classes (0–4) for comparison and to investigate which classes are hardest and easiest to detect. The dataset split of training, validation, and test datasets for five NHI classes is explained previously in the data material section Table 1c, and the corresponding grouped class frequencies for Type A and Type B, obtained by aggregating these counts, are provided in Supplementary Table 4.

Each WSI is represented as a bag of non-overlapping patch embeddings. Because bag sizes vary across slides, bags were zero-padded to the maximum length within each mini-batch and accompanied by a padding mask (1 = real patch, 0 = pad). Training was done using the Adam optimizer with a learning rate of 1 ×10^−5^ and focal loss with epoch-wise dynamic class weights to mitigate class imbalance. Dropout was applied in the encoder to reduce overfitting. Model and parameter selection were based on validation performance, and the final results were reported on the independent test set using the best checkpoint (early stopping).

### Ablation study of region filtering

To evaluate the impact of region filtering, we performed an ablation study comparing REMIL-IBD with and without the Cerberus-based filtering stage. In the filtered setting, Cerberus was used to identify informative tissue regions, and only patches retained by this step were passed to the foundation-model encoder and MIL classifier. In the unfiltered setting, all non-background patches were processed directly. This design allowed us to quantify the effects of filtering on pipeline runtime and classification performance.

## Results

Cerberus-derived annotation maps were reviewed by an experienced gastrointestinal pathologist. At the cellular level, occasional misclassifications of neutrophils as lymphocytes were observed, whereas the tissue-level segmentation was judged to be reliable. Accordingly, only the tissue segmentation maps were used to filter patches for the REMIL-IBD experiments, and cell-level prediction maps were not used as input features.

### FM-region baseline statistics reproduces clinical observation

**Fig. 3:**
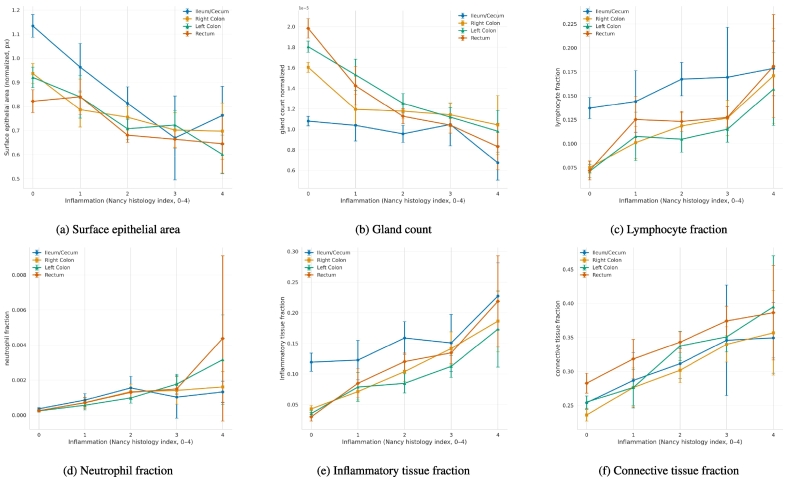
Results from FM-Region statistics baseline. Changes in FM-Region baseline statistics extracted from Cerberus output across all the locations and five inflammation severity (NHI) are shown in the figure. Metrics include gland and epithelial integrity proxies (a,b), and immune cell densities (c,d), tissue type area fractions (e,f). These distinguish key pathological hallmarks of IBD, such as reduced surface epithelial area (a) and gland count (b), gradually increasing lymphocyte (c) and neutrophil fraction (d), increased inflamed (e), and connective (f) tissue fractions with increasing disease severity, irrespective of the biopsy location.

Quantitative histology metrics from Cerberus confirmed expected NHI-dependent changes: gland count and surface epithelial area decreased, whereas inflamed and connective tissue fractions increased with higher grades (Fig. 3). The lymphocyte fraction showed a baseline presence that gradually increased, whereas the neutrophil fraction remained low at NHI 0–2 and rose sharply at NHI 3–4.

### Result from gradient boost classification experiment

The performance of the GB classification experiment based on statistical features is presented in Table 2a, and b. The GB baseline was used to assess whether slide-level FM-region statistics features derived from Cerberus outputs contain sufficient information to discriminate inflammation severity without spatial context.

**Table 2:** Performance of gradient boosting (GB) baseline and REMIL-IBD models (UNI, Virchow2, Cerberus embeddings) under Type A and Type B grouping schemes.

*(a) Type A grouping (0–1, 2, 3–4)*				

Metric	GB	UNI	Virchow2	Cerberus

Accuracy	0.71	0.834	**0.842**	0.762
Macro F1	0.64	0.780	**0.787**	0.692
Weighted F1	0.71	0.832	**0.839**	0.757
F1-score per class				
No active colitis	0.85	0.924	**0.927**	0.892
Mild active colitis	0.53	0.727	**0.748**	0.563
Severe active colitis	0.56	**0.693**	0.685	0.622
AUC per class (HFMs only)				
No active colitis	–	0.94	**0.95**	0.93
Mild active colitis	–	0.86	**0.88**	0.81
Severe active colitis	–	**0.95**	**0.95**	0.93

(b) Type B grouping (0, 1–2, 3–4)				

Metric	GB	UNI	Virchow2	Cerberus

Accuracy	0.74	**0.826**	**0.826**	0.717
Macro F1	0.69	0.788	**0.799**	0.689
Weighted F1	0.74	**0.825**	0.824	0.720
F1-score per class				
No disease	0.82	**0.921**	0.906	0.842
Low-grade Inflammation	0.69	**0.768**	0.760	0.615
Severe active colitis	0.61	0.675	**0.732**	0.611
AUC per class (HFMs only)				
No disease	–	**0.96**	**0.96**	0.93
Low-grade inflammation	–	**0.88**	**0.88**	0.80
Severe active colitis	–	0.95	**0.96**	0.92

In the binary classification setup (inactive vs active IBD), the GB classifier achieved an accuracy of 0.83. In the three-class classification task, the model achieved an overall accuracy ranging from 0.71 to 0.74 (Table 2).

These results confirm that FM-region statistical features capture meaningful disease-related signals, such as increased neutrophil presence at NHI 3–4 and glandular depletion, supporting their biological interpretability. In addition, the segmentation outputs themselves provide quantitative tissue summaries and visual cell and tissue maps that can be inspected independently of the classification models. However, because these statistical summaries do not retain spatial relationships between tissue structures, methods that incorporate spatial context, like REMIL-IBD, provide additional discriminatory information for inflammation severity.

### Results from REMIL-IBD classification

This experiment corresponds to row 2 in Fig. 1. The REMIL-IBD models (REMIL-IBD-Cerberus, REMIL-IBD-Virchow2, and REMIL-IBD-UNI) were trained using feature embeddings from three general-purpose HFMs (UNI, Virchow2, and Cerberus) to perform three-class inflammation grading. No task-specific fine-tuning was applied to the foundation models; training was restricted to the ABMIL parameters. To compare which foundation model provides the most informative feature representations, all components were kept constant except HFM choice and embedding dimensionality.

**Fig. 4:**
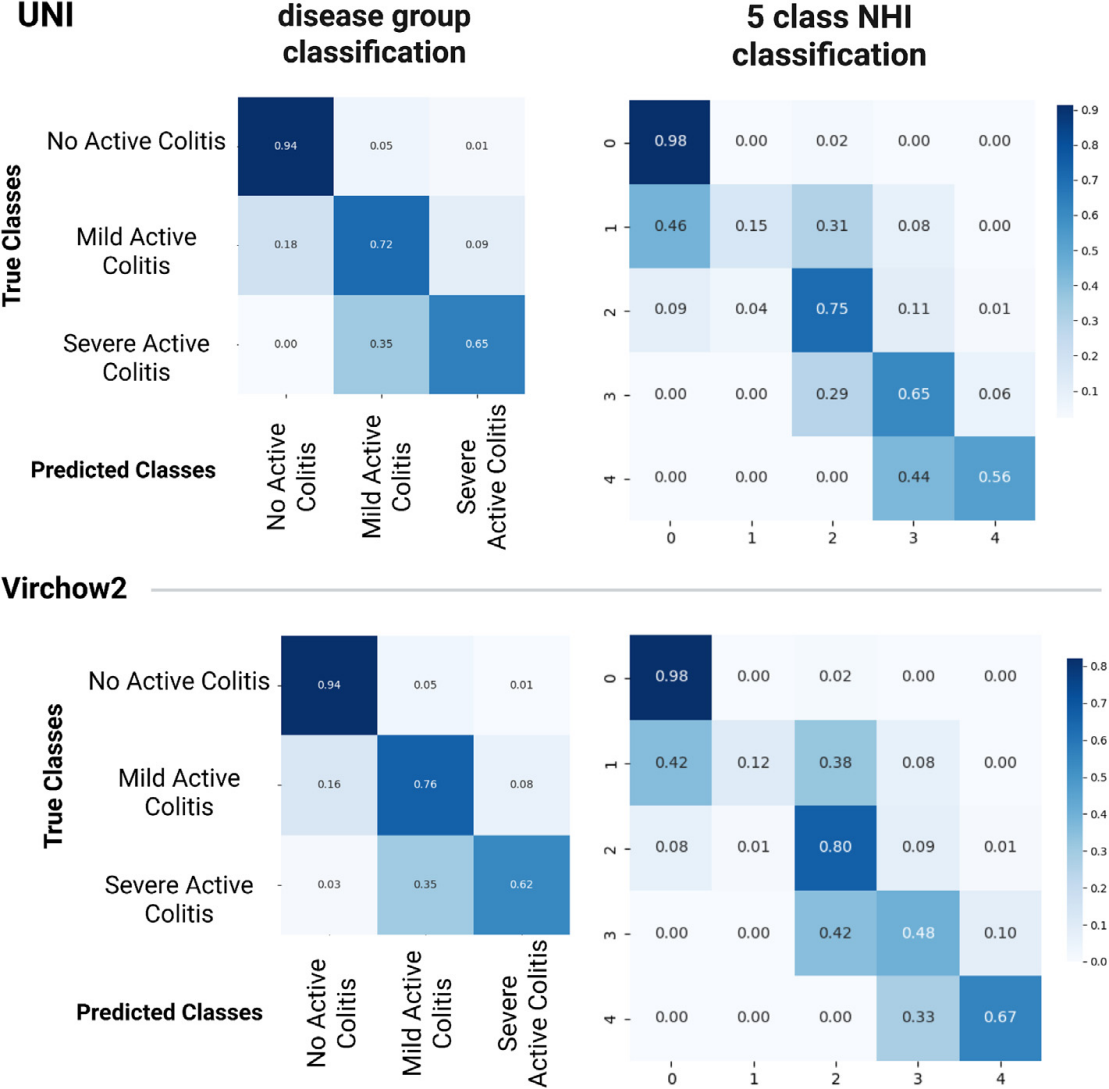
Confusion matrices summarizing REMIL-IBD model performance for three and five disease severity classes across all the best-performing models.

We evaluated both grouping strategies, Type A and Type B, in the *three-class* setting (Table 2a and b). For clarity, Type A was retained as the primary label formulation in the remainder of the manuscript, because the underlying workflow does not change and only the class grouping differs. Across both grouping strategies, UNI and Virchow2 consistently achieved the strongest overall performance (accuracy = 0.83–0.84), whereas Cerberus performed lower overall. Type B remained competitive with Type A and showed a modest improvement in Macro F1 with most balanced performance, whereas accuracy and weighted F1 were broadly similar or slightly favored Type A, depending on the model. In both grouping schemes, the intermediate class remained the most difficult to classify, whereas the extreme classes showed the highest F1 scores. Confusion matrices (See Fig. 4) confirm that most misclassifications were concentrated in the intermediate class.

In the next experiment, we train the ABMIL-based classifier to predict the complete *five-class* Nancy index (NHI 0–4) using feature embeddings extracted from the HFMs, allowing comparison with three-class grouping strategies. Without fine-tuning the foundation models, training was restricted to the ABMIL parameters. Performance metrics are shown in Table 3a and confusion metrics in last column of Fig. 4.

**Table 3:** Subtable 3a (*Left*) Overall and per-class AUC and F1 scores for five-class NHI grading using REMIL-IBD models. AUC reflects discriminative ability between classes, and F1 indicates balance between precision and recall for each class. Subtable 3b (*Right*) Per-class and Macro F1 scores for five-class and three-class NHI classification with and without region filtering for Virchow2 and UNI. HR = histological remission, MHA = mild histological activity, AD = active disease. Bold values indicate the best result per row.

*(a) Overall and per-class performance for five-class NHI grading using REMIL-IBD models*			

Metrics	UNI	Virchow2	Cerberus

Overall			
Accuracy	**0.777**	**0.777**	0.577
Macro F1	**0.621**	0.606	0.390
Weighted F1	**0.755**	0.748	0.580
Per-class (AUC, F1)			
NHI 0	AUC: **0.96**	AUC: 0.95	AUC: 0.93
	F1: 0.92	F1: **0.93**	F1: 0.83
NHI 1	AUC: 0.69	AUC: 0.69	AUC: 0.69
	F1: **0.24**	F1: 0.20	F1: 0.06
NHI 2	AUC: 0.89	AUC: **0.90**	AUC: 0.77
	F1: 0.75	F1: **0.75**	F1: 0.47
NHI 3	AUC: **0.93**	AUC: **0.93**	AUC: 0.85
	F1: **0.62**	F1: 0.52	F1: 0.41
NHI 4	AUC: **0.98**	AUC: **0.98**	AUC: 0.92
	F1: 0.59	F1: **0.63**	F1: 0.18


*(b) Effect of region filtering on performance of REMIL-IBD classification experiment for Virchow2 and UNI*				

	Virchow2		UNI	
	Unfiltered	Filtered	Unfiltered	Filtered

5-class NHI				
NHI 0	0.889	**0.927**	0.903	**0.915**
NHI 1	0.188	**0.200**	**0.261**	0.235
NHI 2	0.717	**0.750**	0.658	**0.731**
NHI 3	0.444	**0.508**	0.533	**0.571**
NHI 4	0.526	**0.600**	0.526	**0.588**
Accuracy	0.730	**0.770**	0.730	**0.766**
Macro F1	0.550	**0.597**	0.576	**0.610**
Weighted F1	0.706	**0.745**	0.714	**0.744**
3-class NHI				
HR	0.885	0.906	0.919	0.908
MHA	**0.650**	0.615	0.676	**0.709**
AD	0.650	**0.660**	0.690	**0.701**
Accuracy	0.777	**0.781**	0.815	**0.819**
Macro F1	0.728	0.727	0.762	**0.773**
Weighted F1	0.789	0.785	0.815	0.820

Overall performance was lower than in the three-class setting, reflecting the increased difficulty of fine-grained grading, with UNI and Virchow2 still achieving accuracies around 0.78 and Macro F1s around 0.62, whereas Cerberus showed clearly lower and less stable performance. NHI 0 and NHI 4 were the most consistently classified classes, with both UNI and Virchow2 achieving high F1 scores (>0.90) for these extreme classes. In contrast, NHI 1 showed the lowest F1 scores (≤0.25) across all models and was frequently confused with NHI 2. Intermediate classes NHI = 2 and 3 also showed overlap with adjacent grades, as reflected in the confusion matrix. AUC showed separability was high for extreme cases with AUC above 0.9, and was much weaker for the intermediate grades (AUC in the 0.6–0.7 range). Weighted-F1 values were higher than Macro F1 across all models, as performance was driven in part by the better classification of the more frequent and consistently predicted classes (NHI 0 and NHI 4).

### Result from region filtering ablation study

Region filtering introduced an additional preprocessing stage, requiring Cerberus tissue segmentation before patch selection and adding approximately 25 min per WSI. Region filtering reduced 38% of the input patches for the MIL, which in turn reduced feature extraction and input preparation time by 26–28%. The reduction in patch count also resulted in shorter MIL training epochs. Full runtime comparisons across pipeline stages are provided in the Table B.1. These findings indicate that although Cerberus segmentation introduces an upfront per-WSI cost, it substantially reduces the computational burden of all subsequent stages. Nevertheless, the total processing time per slide, including the region filtering step, remained longer than in the unfiltered pipeline.

At the same time, region filtering consistently improved classification performance for both Virchow2 and UNI. Although the overall accuracy was comparable due to the dominant class NHI-0, the most consistent and significant class-level gains were observed for the higher-severity grades (NHI ≥ 2). Although NHI-1 remained the most difficult class and showed only marginal changes after filtering, consistent with its clinical ambiguity as a transitional grade. Full class-wise results are provided in Table 3b. A similar pattern was also observed in the three-class setting. Whereas overall performance changed minimally, the Active Disease class prediction improved with filtering, mirroring the gains seen at higher NHI severity in the five-class task, and the intermediate class remained inconclusive, similar to the ambiguous NHI-1 grade.

The ablation study also revealed that the benefit of region filtering depends strongly on whether it is applied consistently across both training and inference. When filtering was used throughout the entire pipeline (condition A), performance was best overall, with the most notable class-level gains in NHI 3 and NHI 4. Applying filtering only at inference time on a model trained without filtering (condition C) yielded intermediate performance, whereas filtering only during training and evaluating on unfiltered patches (condition B) produced the weakest results for Virchow2, falling below the fully unfiltered baseline (condition D). NHI 1 remained the most difficult class to classify, regardless of the filtering condition, in both models. In the three disease-state classification, no single filtering condition consistently dominated across all classes and metrics, suggesting that region-level filtering has a limited effect on the three-class grouping, whether applied during training or inference. Full class-wise results for all four conditions are provided in Table B.2.

### Attention mechanisms and informative regions

To investigate where the model looks at when forming slide-level three-class disease state predictions, we visualized attention score distribution from UNI and Virchow2-based REMIL-IBD models, offering interpretability of model predictions. For illustration, we focus on Virchow2 and the type A grouping experiment, whereas detailed examples for both models across grouping strategies are provided in the Supplementary material (Figs. 1–6 in the Supplementary Material).

**Fig. 5:**
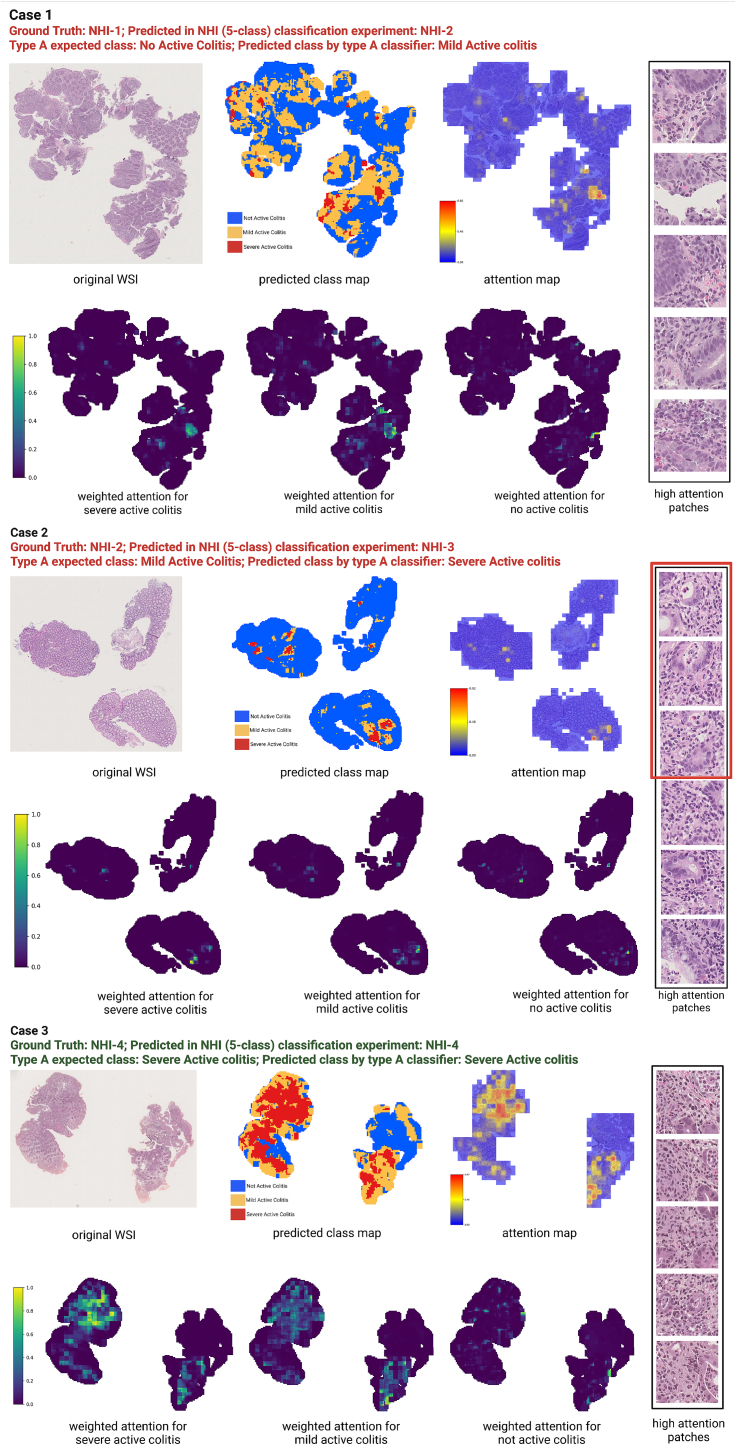
Representative attention maps from the REMIL-IBD-Virchow2 model for three NHI cases spanning the inflammation spectrum. From top to bottom: Patient case 1 (NHI-1), Patient case 2 (NHI-2), and Patient case 3 (NHI-4). For each case, the original WSI, slide-level prediction map, attention map, and representative high-attention patches are shown for the Type A grouping scheme.

Three representative cases from different areas of the inflammation spectrum, NHI 0–4, are used to showcase how, with increasing inflammation, the histological patterns the model prioritizes change (See Fig. 5).

*Patient case 1.* The NHI-1 biopsy showed attention concentrated in a limited mucosal region, with the highest-weighted patches corresponding to mildly abnormal epithelium with distorted crypt architecture, slightly higher inflammatory cell density in the lamina propria with occasional neutrophil, representing mildly inflamed or borderline normal biopsy.

*Patient case 2.* Although was manually graded as NHI-2, the high attention patches included focal crypt distortion and rare crypt abscesses (patches highlighted in right panel of Fig. 5), and the strongest signals again appeared in the higher-inflammation channels.

*Patient case 3.* The NHI-4 biopsy showed broadly distributed high attention across severely affected mucosa, with high-weighted patches highlighting dense inflammatory infiltrates, crypt abscesses, and epithelial erosion.

Across these examples, lower-grade inflammation was typically associated with more localized or focal attention, whereas higher-grade inflammation showed broader and more spatially extensive attention across the mucosa. In all three cases, the strongest attention weights were generally assigned to higher-inflammation channels, and the highlighted regions were concentrated in histologically abnormal tissue rather than distributed uniformly across the slide.

## Discussion

In this study, HFMs pretrained with extensive cell and tissue-level annotations, such as Cerberus, provided a practical means to capture inflammatory patterns without requiring exhaustive manual annotation by pathologists. Even simple region-level statistics derived from these features already capture sufficient information to reproduce clinically plausible progressive changes across the NHI spectrum, such as gland loss, epithelial damage, and increasing inflammatory and stromal components, that are cumbersome to quantify manually. The GB classifier served as a first test, indicating that these segmentation-derived statistical summaries support slide-level inflammation grading.

Beyond classification performance, the FM-region statistics baseline serves an important clinical purpose: it provides a quantitative profile for each WSI that summarizes tissue status in human-readable terms. For a new test case, this profile directly shows the extent of epithelial erosion, relative gland depletion, neutrophil and lymphocyte infiltration density, and stromal expansion, each of which is recognized as a histopathological hallmark of IBD progression. Unlike attention maps, this not only makes it a biologically meaningful severity classifier but also a WSI-level summary tool that does not require pathologists to be familiar with ML inferences at test time.

Compared to the tree-based FM-Region baseline, the REMIL models (REMIL-IBD-UNI and REMIL-IBD-Virchow2) achieved higher accuracy and more stable predictions across folds. This indicates that the attention-mediated MIL framework using frozen foundation-model embeddings supports accurate slide-level grading when only the top layers of the MIL framework are trained. The selection of tissue types used for region filtering was guided by expected histopathological relevance and supported by preliminary exploratory experiments. Future work will systematically evaluate alternative combinations of tissue types, similar to prior work,^28^ to further enhance interpretability by identifying diagnostically important tissue classes that complement the spatial localization provided by attention-based heatmaps.

REMIL-IBD, with lightweight MIL classifier layer training, can already distinguish clear-cut inflammatory states surprisingly well, but subtle changes in the intermediate stages test its representational limits. In our added comparison, Type B showed a modest improvement in Macro F1, suggesting that separating healthy tissue from any inflammation may provide a slightly more balanced learning signal, whereas accuracy and weighted F1 remained comparable or slightly favored Type A in some models. We therefore present Type B as a biologically motivated alternative grouping that is competitive with Type A rather than strictly superior.

Despite this overall good performance, a central observation in all the experiments is that errors are concentrated around the intermediate NHI grades rather than at the extremes. Both tree-based baselines and REMIL-IBD models had difficulty with NHI-1 and 2, which were frequently confused with higher or lower grades. This pattern mirrors clinical experience: NHI-1 is defined by the absence of ulceration and an acute infiltrate, but by moderate-to-severe chronic inflammation,^43^ which is inherently more subjective than histological remission or clearly severe disease and therefore more vulnerable to interobserver variability and borderline morphology. Previous reports of lower agreement for NHI-1 and only moderate overall reliability of the NHI system suggest that noisy training label data, rather than model capacity, are likely the main performance constraints.^1^ In this context, misclassifications likely reflect both label ambiguity and the inherent difficulty of defining sharp boundaries between adjacent severity levels, and attention maps can help in interpreting where the underlying grading system is most difficult to apply, not merely where the algorithms “fail.”

The ablation study showed that Cerberus-based region filtering adds significant value to the REMIL-IBD pipeline beyond a conventional unfiltered MIL approach. Although filtering introduced an upfront Cerberus segmentation stage, this was offset by reduced downstream computation and, more importantly, consistently improved classification of higher-severity inflammation grades, particularly NHI 3 and NHI 4 in the five-class setting and active disease in the three-class setting, which are most critical for guiding treatment-escalation decisions. This consistent advantage of filtering for active inflammation detection across both granularities suggests that without filtering, downstream MIL models trained on weakly labeled WSIs are exposed to non-informative patches that dilute the signal from diagnostically relevant tissue regions. The ablation further showed that this benefit was conditional on consistent application: MIL models trained and evaluated on filtered patches outperformed all other configurations, whereas mismatched filtering between training and inference degraded performance below the fully unfiltered baseline. Region filtering is therefore not simply a preprocessing convenience but a pipeline design decision that should be applied uniformly across training and deployment to avoid introducing distribution shift. This sensitivity was specific to the fine-grained five-class task; the three-class NHI grouping showed minimal variation across all four conditions (<2% in accuracy and Macro F1) across all configurations, suggesting that coarser disease categories are more easily separable from patch-level features and less susceptible to patch-level noise.

Complementing these quantitative findings, inspection of attention maps across representative cases provided qualitative evidence that the models focus on histologically meaningful regions. High-attention patches at both ends of the inflammation severity scale showed expected histological patterns: quiescent mucosa with localized attention in the lower inflammation state and dense inflammatory infiltrates, neutrophils, cryptitis, crypt abscesses, and epithelial erosion with a broader and more diffuse attention pattern in the higher inflammation state. This supports the face validity of the learned representations. Attention also showed the model's potential sensitivity to focal severe histological features within otherwise intermediate-grade biopsies. In Patient case 2, the highlighted regions included crypt abscesses and cryptitis despite the overall NHI-2 label. This suggests that sparse but diagnostically important lesions can strongly influence slide-level prediction, highlighting the efficiency of attention-based models for identifying localized disease activity, and improving consensus annotation of such borderline difficult cases may be more beneficial than further increasing model complexity or size.

The results of the study indicate that current HFMs already capture much of the multiscale information needed for clinically meaningful inflammation grading, and that relatively simple downstream architectures, such as the REMIL-IBD pipeline and gradient-boosted trees, can effectively exploit these features. Taken together, the results support HFMs as a strong, general-purpose substrate for IBD histopathology, whereas emphasizing that consensus in annotation practice and task formulation may be at least as important as further architectural innovation.

### Limitations

The study is constrained by limited sample sizes in some extreme categories, and the lack of external validation on independent cohorts with diverse scanner platforms and staining protocols, as a consequence of the absence of publicly available slide-level NHI-annotated datasets. Although REMIL-IBD works very well in the current cohort, we cannot comment on its generalizability. Future studies should therefore evaluate generalizability across independent cohorts and examine whether alternative tissue-type filtering strategies or improved annotation protocols can further improve robustness. In addition, NHI was originally validated for UC colonic biopsies; however, in the absence of a CD-specific histological activity index, it was also applied to colonic CD samples here, in line with previous reports and expert practice.^44^, ^45^, ^46^, ^47^ This cross-application remains an inherent limitation of the present study for CD samples.

## Conclusion and future work

We present a fully automated pipeline, REMIL-IBD, for WSI interpretation of IBD biopsies, offering region-of-interest (ROI) extraction, quantitative histological feature measurements, and classification of inflammation grade with an accuracy of 0.83 and AUC values of 0.96 for no disease and 0.95 for severe active colitis. With this work, HFMs such as Cerberus, pretrained on large datasets, are shown to be useful for tissue and feature segmentation in IBD, providing meaningful delineation of ROIs and cell types. This facilitates counting and measuring cells and regions that would not have been feasible to perform manually due to the substantial workload. Summary statistics from this automatic segmentation and counting correlate with the inflammation grade as expected. In addition, general-purpose HFMs such as UNI and Virchow2 can be used to derive feature embeddings from automatically extracted ROIs. A lightweight gated-attention MIL setup with a final classifier layer, as implemented in REMIL-IBD, yields promising automatic classification of IBD inflammation grade and offers interpretability through classification maps and attention maps.

Future work would explore prototype-based MIL for human-guided refinement,^48^ improving consensus annotation for challenging intermediate grades, integrating HFM features with clinical and molecular data in multimodal frameworks, and validating across multi-center cohorts with diverse scanners and staining protocols.

## Declaration of generative AI and AI-assisted technologies in the manuscript preparation process

During the preparation of this work, the authors used Writefull and ChatGPT in order to improve grammar and language clarity, and also for code debugging. After using this tool/service, the authors reviewed and edited the content as needed and take full responsibility for the content of the published article.

## Ethics statement and patient consent

We used samples from the Stavanger University Hospital IBD (SUSI) (NCT01551563) project, permitted by the Norwegian Regional Ethics Committee (REK 2011*/*2631), and written informed consent was obtained from all participants.

## CRediT authorship contribution statement

A.B., K.E., M.v.d.G., and E.J. conceived and designed the study. A.B. and K.E. implemented methods, conducted experiments and analyses, and drafted the manuscript, with K.E. supervising technical work and result interpretation. O.G.A. and T.G. provided clinical*/*pathological expertise and critical revisions. All authors contributed to discussion, study design, revisions, and approved the final manuscript.

## Declaration of competing interest

The authors declare the following financial interests/personal relationships which may be considered as potential competing interests:

Tore Grimstad reports a relationship with Ferring Pharmaceuticals that includes: speaking and lecture fees. Tore Grimstad reports a relationship with Takeda AS that includes: consulting or advisory. Tore Grimstad reports a relationship with Galapagos that includes: consulting or advisory. Tore Grimstad reports a relationship with Johnson & Johnson that includes: consulting or advisory. Emiel Janssen reports a relationship with AbbVie AS that includes: funding grants. Emiel Janssen reports a relationship with Tillotts Pharma AB that includes: funding grants. If there are other authors, they declare that they have no known competing financial interests or personal relationships that could have appeared to influence the work reported in this article.

## Figures and Tables

**Table B.1:** Runtime comparison of the REMIL-IBD pipeline with and without region filtering (runtime is comparable for both five-class and three-class classification experiment). Values are mean ± standard deviation per WSI unless otherwise stated.

Stage	Variant	Without filtering	With filtering	Change in runtime
Tissue detection (s)		73±40	73±40	0.0 s
Cerberus segmentation (s)		–	1470±1022	–
Input preparation for ABMIL (s)		73±39	54±33	−19 s
Mean patches per WSI		2128	1336	−792 s
	UNI	223±110	159±90	−64 s
Feature extraction (s)	Virchow2	438±220	312±174	−126 s
	Cerberus	133±12	124±5	−9 s
MIL runtime per epoch (for 1224 train sample) (min)^a^	UNI	67	43	−24 min
	Virchow2	52	47	−5 min
	Cerberus	54	41	−13 min
Apprx. MIL training time per WSI (s)^a^	UNI	3.3	2.1	−1.2 s
	Virchow2	2.6	2.3	−0.3 s
	Cerberus	2.7	2	−0.7 s
Per WSI appx. training time change due to region filtering (s)	UNI	–	–	+1386 s
	Virchow2	–	–	+1325 s
	Cerberus	–	–	+1441 s

a MIL runtime is reported per training epoch across all training slides, not per WSI.

**Table B.2:** Class-wise F1 scores for the 2 × 2 ablation study across Virchow2 and UNI. Columns are ordered from (D, train = unfiltered and test = unfiltered; C, train = unfiltered and test = filtered; B, train = filtered and test = unfiltered; A, train = filtered and test = filtered). Best values per class across the four conditions are shown in bold.

	*(a) Class-wise F1 scores for the 2 ×* *2 ablation study across Virchow2 and UNI for 5 class NHI classification*							
	Virchow2				UNI			
Class	D	C	B	A	D	C	B	A

0	0.8889	0.8898	0.8996	**0.9272**	0.9035	0.8907	0.9064	**0.9154**
1	0.1875	**0.2222**	0.0000	0.2000	**0.2609**	0.2500	0.1379	0.2353
2	0.7168	0.7429	0.7239	**0.7500**	0.6575	0.6803	**0.7389**	0.7308
3	0.4444	0.5185	0.4231	**0.5085**	0.5333	0.5574	0.5333	**0.5714**
4	0.5263	**0.6000**	**0.6000**	**0.6000**	0.5263	**0.6316**	0.5882	0.5882
Accuracy	0.7321	0.7472	0.7434	**0.7736**	0.7245	0.7170	0.7623	**0.7660**
Macro F1	0.5528	0.5947	0.5293	**0.5971**	0.5763	0.6020	0.5809	**0.6082**
Weighted F1	0.7064	0.7289	0.6950	**0.7449**	0.7138	0.7197	0.7285	**0.7444**

	*(b) Class-wise F1 scores for the 2 ×* *2 ablation study across Virchow2 and UNI for 3-class NHI grading*							
	Virchow2				UNI			
Class	D	C	B	A	D	C	B	A

HR	0.8850	0.8958	**0.9097**	0.9059	**0.9195**	0.9085	0.9139	0.9085
MHA	0.6503	**0.6625**	0.5669	0.6154	0.6759	0.6757	0.7059	**0.7089**
AD	0.6500	0.6341	0.6452	**0.6600**	0.6897	**0.7126**	0.6933	0.7013
Accuracy	0.7774	**0.7849**	0.7811	0.7811	0.8151	0.8113	**0.8226**	0.8189
Macro F1	0.7284	**0.7308**	0.7073	0.7271	0.7617	0.7656	0.7710	**0.7729**
Weighted F1	0.7822	0.7894	0.7715	**0.7855**	0.8149	0.8122	**0.8210**	0.8200

## Data Availability

The data underlying this study are not publicly available due to ethical and legal restrictions, but may be provided upon reasonable request and subject to approval.

## Appendix A Technical background

In this section, we briefly introduce key concepts from artificial intelligence (AI) employed in REMIL-IBD pipeline, along with the HFMs used.

### A.1 AI in computational pathology

AI in computational pathology refers to deep neural networks (DNNs), which are a subset of machine learning (ML), which is the study of methods for learning a function from data. The basic building blocks of a DNN algorithm can be divided into: (1) A neural network architecture with trainable parameters, θ. (2) Training data *x_i_*, possibly with truth labels *y_i_*. (3) A loss (objective) function and an optimization algorithm. (4) A learning strategy. The objective is to learn a function, $f_{\theta }\left(\cdot \right)$, that represents the relation between *x* and *y* using the loss function to optimize for θ so that $\hat{y}$ is the approximation of *y*:

(A.1) $$\hat{y}=f_{\theta }\left(x\right).$$

The architectures used in this work are variants of convolutional neural networks (CNNs) and Vision Transformers (ViTs). The most straightforward learning strategy is supervised learning, where labels *y_i_* are available for all data instances *x_i_* in the training set.

### A.2 Multiple instance learning

Multiple instance learning^49^ (MIL) is a weakly supervised learning strategy, where the input data are divided into bags of instances, each bag denoted as $B_{i}=x_{i,1},x_{i,2},\ldots ,x_{i,n_{i}}$ with *n_i_* instances. The model is trained with a label for each bag *y_i_*, rather than for each instance *x_i,j_*, also called weak labels. MIL is a strategy used when only a slide-level diagnosis is available, but lacks region-based labels. Thus, instances *x_i,j_* are image patches, and the bag *B*_*i*_ is the entire WSI (or a part of the WSI). Each instance from a bag is fed through a feature extractor $\varphi_{\theta }$ to produce an embedding $h_{i,j}$.

(A.2) $$h_{i,j}=\varphi_{\theta }\left(x_{i,j}\right).$$

These instance embeddings *h_i,j_* from one bag *B_i_* are then aggregated, and finally the aggregated embedding *z_i_* is passed to a classifier to predict the bag. Early MIL approaches often used simple mean pooling:

(A.3) $$z_{i}=\text{mean}\left(h_{i,1},\ldots ,h_{i,n_{i}}\right)$$

Gated attention-based MIL^50^, ^51^, ^52^ introduced a learnable attention mechanism where each instance embedding $h_{i,j}$ is weighted by an attention score $\alpha_{i,j}$.

(A.4) $$\sum_{j=1}^{n_{i}}\alpha_{i,j}=1,\;\alpha_{i,j}\geq 0$$

(A.5) $$z_{i}=\sum_{j=1}^{n_{i}}\alpha_{i,j}h_{i,j}.$$

The aggregated bag *z_i_* is then passed to a classifier $f_{\psi }$ for a bag prediction $\hat{y}i$.

(A.6) $$\hat{y}i=\mathrm{f\psi}\left(z_{i}\right).$$

The model tries to minimize the loss:

$$\min\theta ,\psi \sum_{i}L\left({\hat{y}}_{i},y_{i}\right)$$

In addition to improved performance, it also gives interpretability, as we can check which patches received the highest attention.

### A.3 Gradient boosting classification

Gradient boosting is an ensemble learning method in which multiple decision trees are trained sequentially, with each new tree correcting the residual errors of the previous ones.^53^ It works on tabular data with independent features $X=x_{1},x_{2},\ldots ,x_{n}$, or possibly embeddings, and a target variable $y=y_{1},y_{2},\ldots ,y_{n}$. The method starts with an initial prediction, typically a constant value such as the mean (for regression)

(A.7) $$F_{0}\left(x\right)=\frac{1}{n}\sum_{i=1}^{n}y_{i}$$

or log-odds (for classification). At each iteration *m*, residuals (the differences between predicted and actual values) are then calculated,

(A.8) $$r_{i}^{\left(m\right)}=y_{i}-F_{m-1}\left(x_{i}\right)$$

and a new tree $h_{m}\left(x\right)$ is fitted to these residuals. The new tree's predictions $h_{m}\left(x\right)$, scaled by a learning rate ν, are added to the previous model's output.

(A.9) $$F_{m}\left(x\right)=F_{m-1}\left(x\right)+\nu \cdot h_{m}\left(x\right).$$

Repeating this process for *M* iterations allows the model to gradually minimize loss and improve accuracy. The final prediction is a sum of all the outputs of all trees in the ensemble:

(A.10) $$F_{M}\left(x\right)=F_{0}\left(x\right)+\nu \sum_{m=1}^{M}h_{m}\left(x\right).$$

### A.4 HFM 1: Cerberus

Cerberus^39^ is a multitask learning (MTL) model designed to segment tissue and classify structures in both H&E- and IMC-stained biopsy images. It has a fully convolutional neural network structure with a shared ResNet34 encoder^54^ that learns common features, multiple independent U-Net-style decoders, and separate loss aggregations for each segmentation task. It's trained on a large colorectal dataset of over 600,000 objects for segmentation and 440,000 patches for classification, with both H&E and immunohistochemical (IHC)-stained tissue images at 20× magnification. Cerberus performs these tasks in an “aligned” manner—meaning they operate on the same tissue type, make predictions for all tasks for that tissue in parallel. This “aligned” approach avoids task entanglement during network optimization raised due to different input types. The Cerberus architecture consists of multiple convolutional layers that extract hierarchical features from input patches, capturing both local and contextual information. The amount of contextual information included is user-defined and determined by the input patch size: larger input patches provide more spatial context but increase computational cost. The output patch size decides the resolution of the segmentation map, with a smaller patch output size giving higher resolution but more computationally intensive predictions. In our pipeline, we used the pretrained Cerberus model as a ready-made annotator for each colon biopsy patch in Section “Nuclei instance classification and tissue segmentation” and the transfer learning ResNet34 encoder as feature extractor in Section “Feature extraction” to produce patch-level feature vectors that serve as inputs to our multiple instance learning classifier in the main manuscript.

### A.5 HFM 2: Virchow2

Virchow2^55^ is a foundation model for Cpath built on a modified DINOv2^56^ self-supervised learning (SSL) algorithm. It uses a Vision Transformer (ViT) architecture, specifically the ViT-H/14 variant, and contains approximately 632 million parameters. It was trained on a wide variety of histopathology datasets across 17 different tissue types, stain protocols (H&E and IHC), and scanned at different resolutions. When provided with non-overlapping 224 × 224 pixel patches as input, the model produces two types of embeddings: first, only the class token; second, the class token concatenated with the mean across all 256 patch tokens (class token + pooled mean). We therefore used the recommended configuration with CLS + mean embeddings (dimension 2560).

### A.6 HFM 3: UNI

UNI (Universal Nucleus Identifier)^41^ is a general-purpose, self-supervised vision encoder specifically tailored for pathology. The model uses the DINOv2 SSL algorithm to pretrain its ViT-Large (ViT-L) architecture. UNI is trained on Mass-100K, one of the largest histology slide collections ever used for SSL. This dataset comprises over 100 million non-overlapping 256 × 256 tissue patches from 100,426 diagnostic H&E-stained WSIs spanning 20 major organ types. UNI's effectiveness was thoroughly assessed across 34 diverse Cpath tasks using different resolution input image patches. When provided with 224 × 224 pixel input patches, it produces a class token embedding of size 1024 for each patch, which later serves as input to a classification model to predict patch-level or slide-level classification.

## Appendix C Supplementary data

Supplementary material containing extended data tables detailing biopsy sampling coverage, disease class distributions across data splits, and GB Boost hyperparameter configurations, alongside comprehensive spatial attention map visualizations for the REMIL-IBD framework (using both UNI and Virchow2 models) across representative clinical cases.
