# Supplementary material for: REMIL-IBD: Region-filtered multiple instance learning for interpretable slide-level grading of inflammatory bowel disease
Source: J Pathol Inform. 2026 May 14;22:100674. doi: 10.1016/j.jpi.2026.100674 (PMC13241852; doi:10.1016/j.jpi.2026.100674)
Supplement: Supplementary file 1 — Supplementary material containing extended data tables detailing biopsy sampling coverage, disease class distributions across data splits, and GB Boost hyperparameter configurations, alongside comprehensive spatial attention map visualizations for the REMIL-IBD framework (using both UNI and Virchow2 models) across representative clinical cases. [file mmc1.pdf]

REMIL-IBD: Region-Filtered Multiple Instance Learning for Interpretable Slide-Level Grading of Inflammatory Bowel Disease

1 Supplementary Figures and Tables

1.1 Supplementary tables from Data Material

Sampling pattern	Ileum/Cecum	Right Colon	Left Colon	Rectum	Count
At least one sample from all 4 regions	✓	✓	✓	✓	368
No ileum/cecum sample	✗	✓	✓	✓	28
Transverse to Rectum only (Limited distal sampling)	✗	✗	✓	✓	6
Transverse/Rectum only	✗	✗	✓	✗	4
Transverse or Rectal missing	✓	✓	✗or ✓	✗or ✓	3
Right or left missing	✓	✗or ✓	✗or ✓	✓	5
Missing rectal sample	✓	✓	✓	✗	23
Cecum + mid colon missing	✗	✓	✗or ✓	✗or ✓	2
Missing region info	-	-	-	-	3

Table 1: Summary of biopsy sampling coverage across intestinal regions.

1.2 Supplementary tables for section 3.1

(a) Type A grouping

Location	Non Active Colitis	Mild Active Colitis	Severe Active Colitis
Ileum and Cecum	297	84	21
Right Colon	267	124	62
Left Colon	232	106	88
Rectum	153	183	126

(b) Type B grouping

Location	No Disease	Low-grade Inflammation	Severe Active Colitis
Ileum and Cecum	270	111	21
Right Colon	223	168	62
Left Colon	197	141	88
Rectum	108	228	126

Table 2: Distribution of disease status across four biopsy locations under Type A and Type B grouping schemes.

1.3 Supplementary tables for section 3.4.1

Hyperparameter	Type A	Type B
Number of trees in the ensemble	200	300
Learning rate (shrinkage factor)	0.05	0.05
Maximum tree depth	3	3
Min. samples to split an internal node	10	15
Min. samples at a leaf node	1	5

Table 3: Hyperparameter used for XGBoost classifiers for Type A and Type B groupings

1.4 Supplementary tables for section 3.5.3

(a) Disease Activity (Type A)

Set	No Active Colitis	Mild Active Colitis	Severe Active Colitis
Training	54.5%	28.6%	16.9%
Validation	52.1%	28.0%	19.8%
Test	56.0%	28.9%	15.1%

(b) Disease Activity (Type B)

Set	No Disease	Low-grade inflammation	Severe Active Colitis
Training	46.2%	36.9%	16.9%
Validation	43.2%	36.9%	19.8%
Test	46.2%	38.7%	15.1%

Table 4: Frequency of different classes across training, validation, and test datasets.

Result

1.5 Analysis of Attention Mechanisms and Informative Regions (Maps for UNI based REMIL-IBD model)

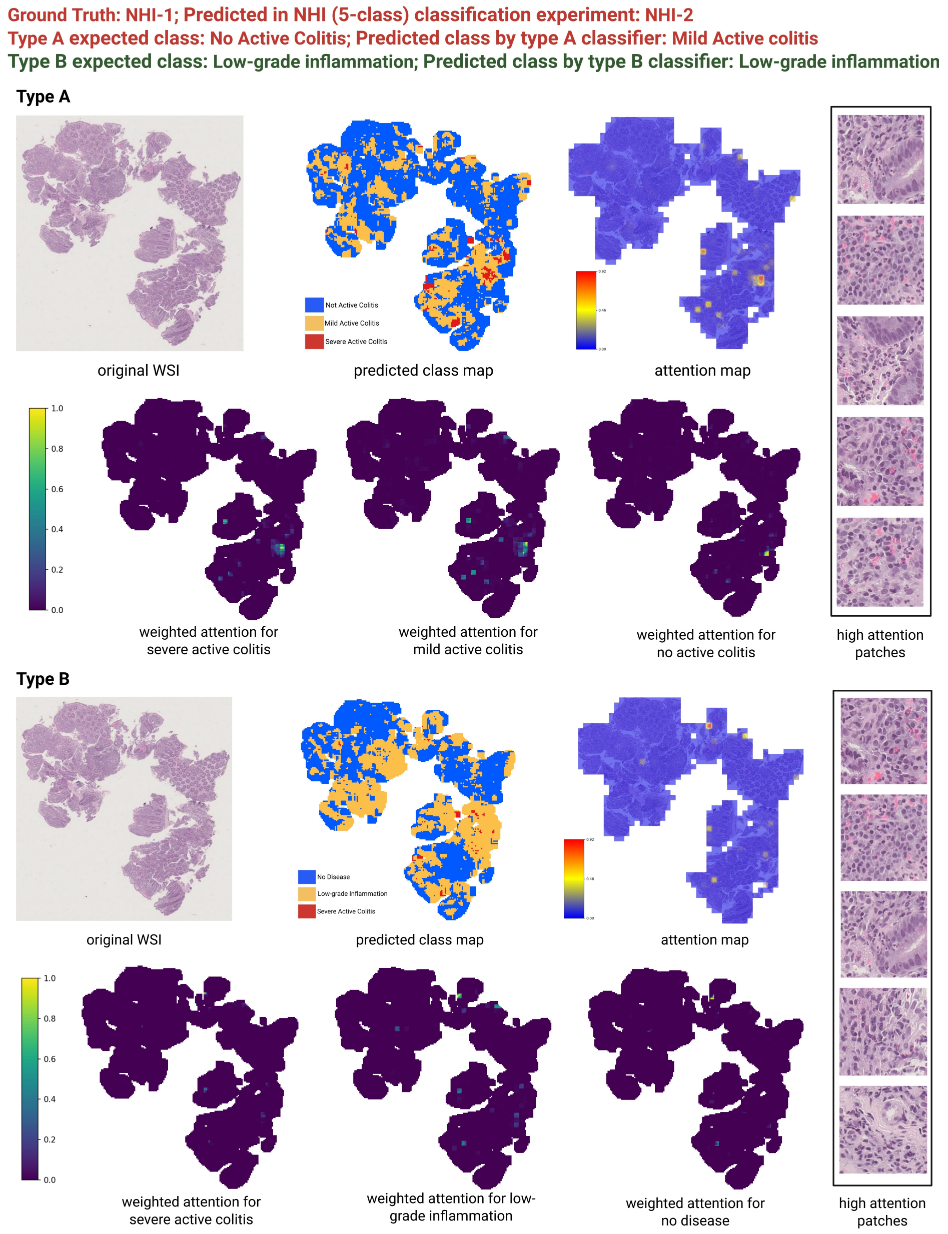

Figure 1: (Case 1) This patient was manually graded as NHI-1, and the 5-class classification predicted NHI-2. Visualization of attention-based predictions for a whole-slide image using the REMIL-IBD-UNI model. The top panel (Type A grouping) shows, from left to right, the original WSI, the corresponding three-class predicted disease map, and the slide-level attention map with representative high-attention patches. For each grouping, class-specific weighted attention maps are displayed beneath the predicted disease map, indicating the spatial distribution of attention for each predicted disease state. The bottom panel (Type B) presents the same sequence of visualizations for the alternative grouping strategy.

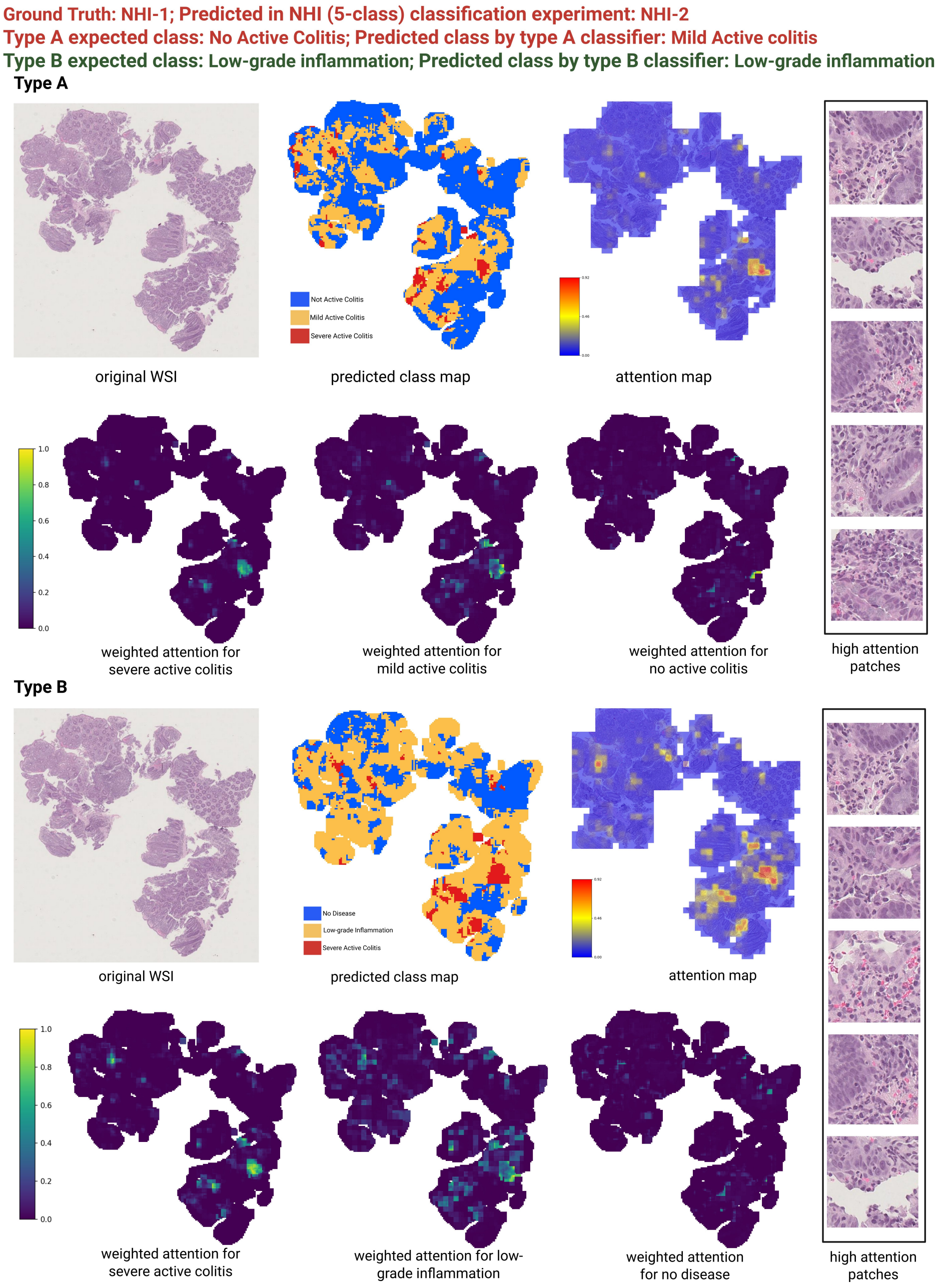

Figure 2: (Case 1) This patient was manually graded as NHI-1, and the 5-class classification predicted NHI-2. Visualization of attention-based predictions for a whole-slide image using the REMIL-IBD-Virchow2 model. Layout same as supplementary figure 1

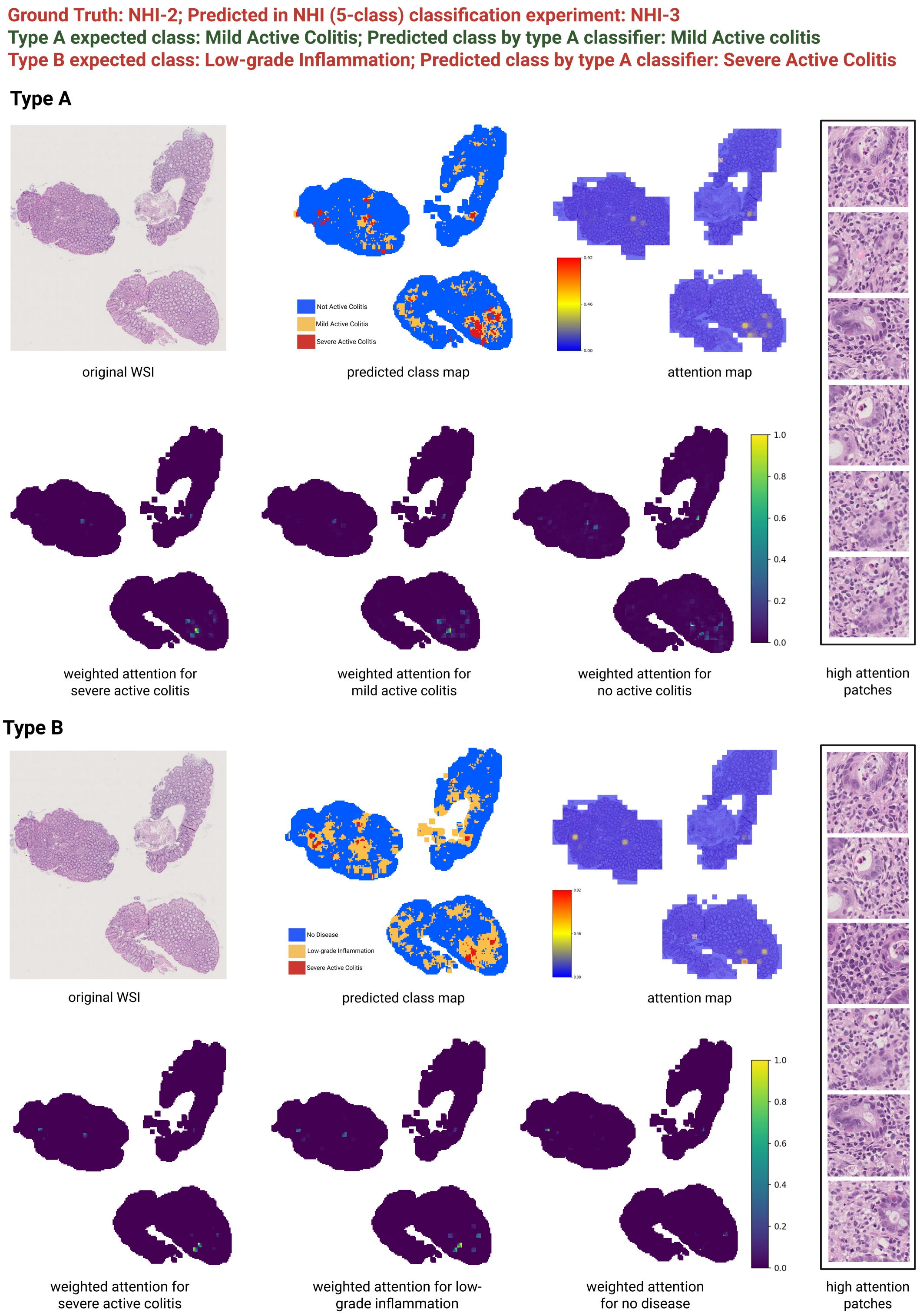

Figure 3: (Case 2) NHI-2 biopsy, manually graded as NHI-2 and predicted as NHI-3 in the 5-class setting. Layout as in supplementary figure 1, using the REMIL-IBD-UNI model.

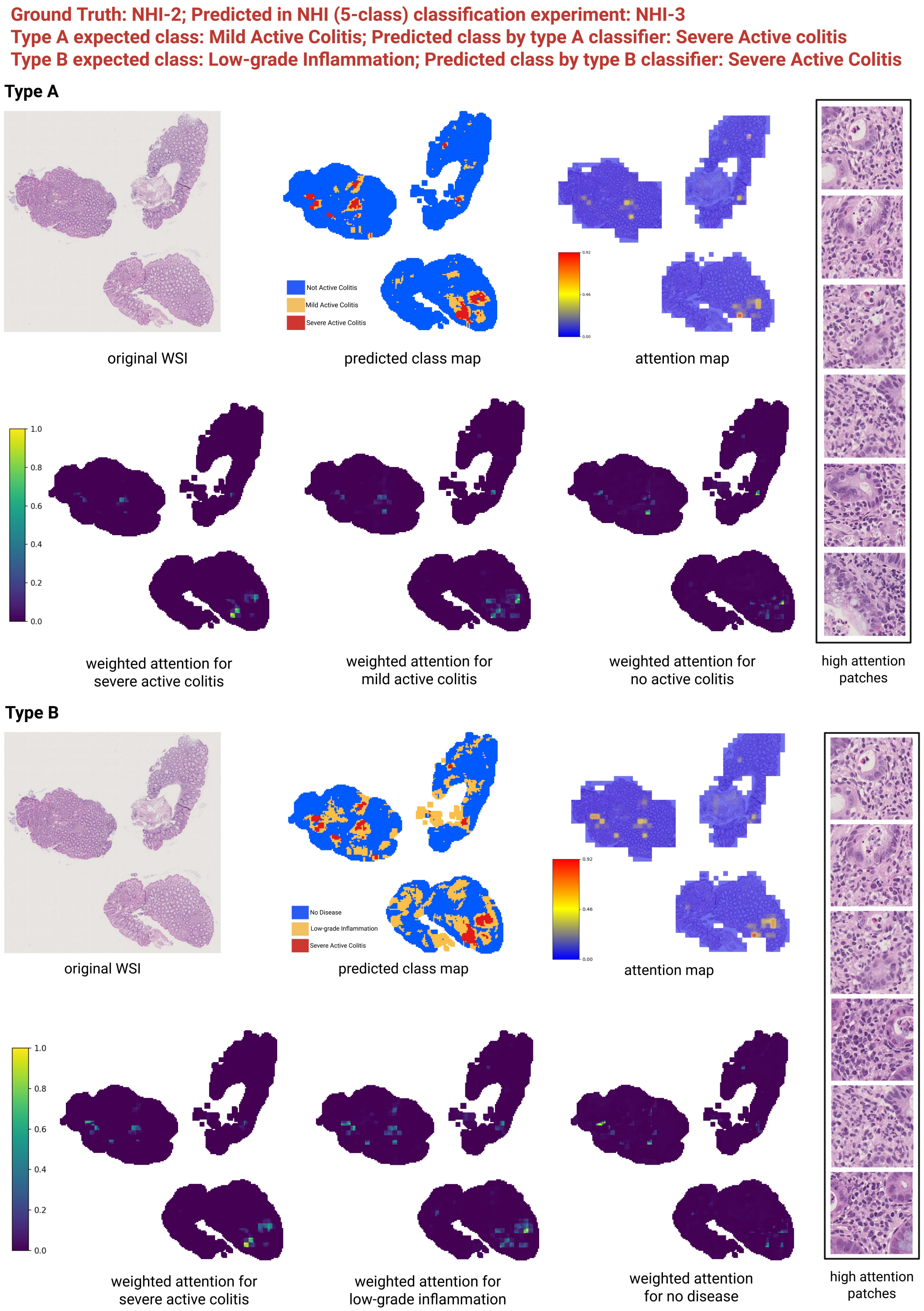

Figure 4: (Case 2) NHI-2 biopsy, manually graded as NHI-2 and predicted as NHI-3 in the 5-class setting. Layout as in supplementary figure 1, using the REMIL-IBD-Virchow2 model.

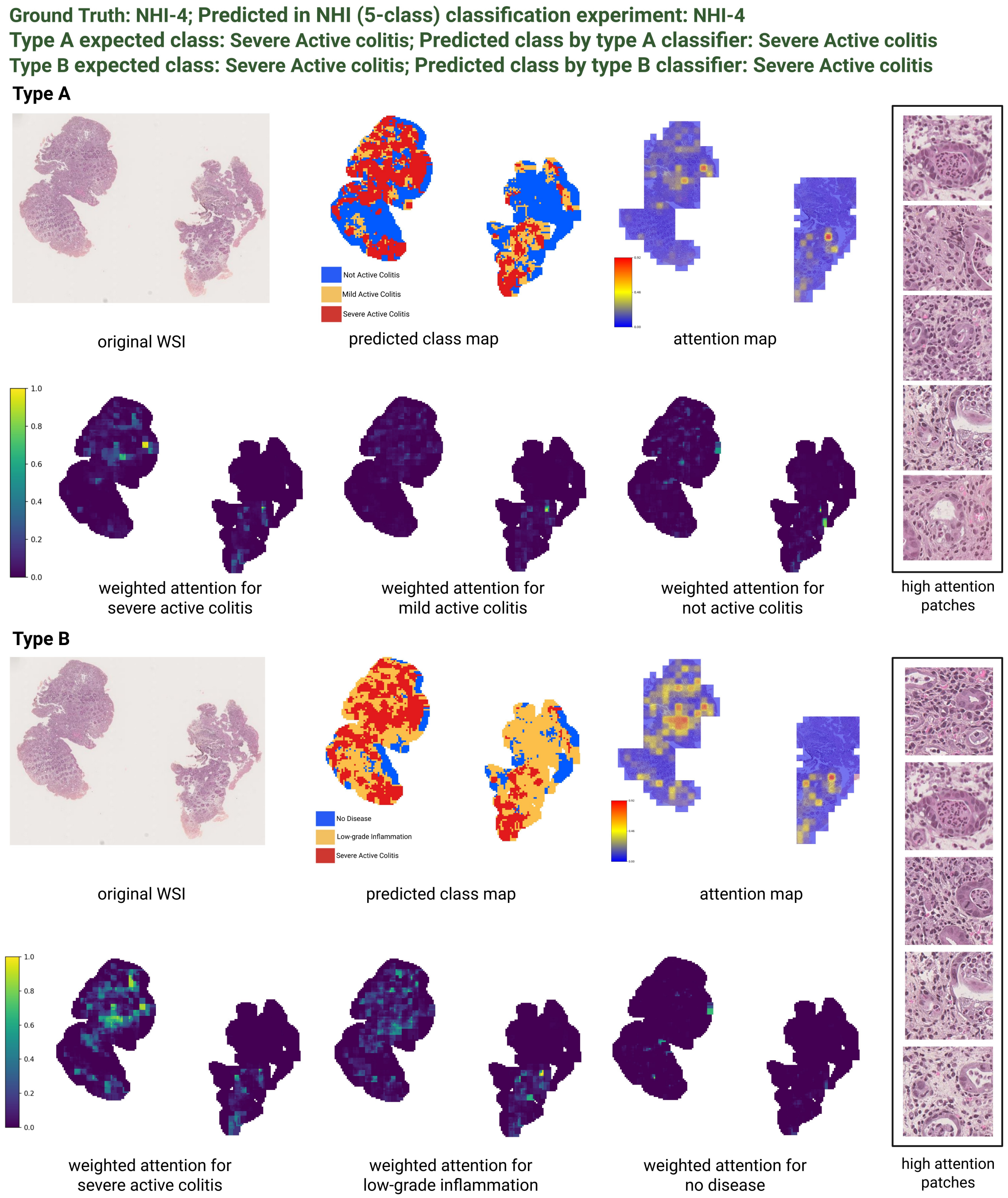

Figure 5: (Case 3) NHI-4 biopsy, manually graded and predicted as NHI-4 in the 5-class setting. Layout as in Figure 1, using the REMIL-IBD-UNI model.

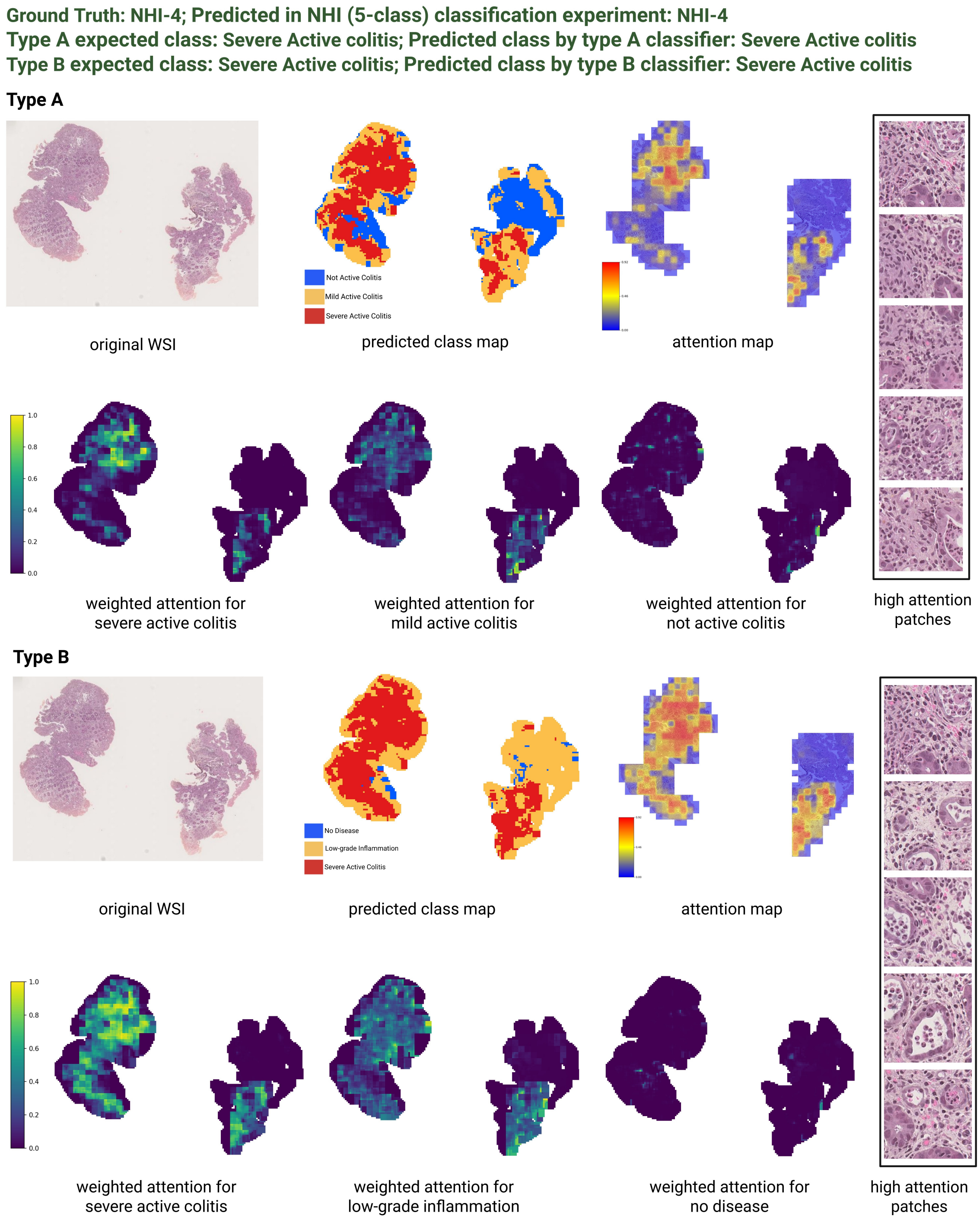

Figure 6: (Case 3) NHI-4 biopsy, manually graded and predicted as NHI-4 in the 5-class setting. Layout as in Figure 1, using the REMIL-IBD-Virchow2 model.
